# Spike protein derived from an apathogenic IBV strain confers attenuated phenotype to a nephropathogenic IBV strain

**DOI:** 10.1128/jvi.01103-26

**Published:** 2026-09-10

**Authors:** James Kirk, Samantha Sives, Adam Rayment, Amber Tappin, Sam Jones, Jessica Miles, Chandana Tennakoon, Elizabeth Martin-Drew, Graham Freimanis, Harry Francis, Thomas P. Peacock, Kate Sutton, Isobel Webb, Andrew D. Davidson, Paul Britton, Erica Bickerton, Sarah Keep

**Affiliations:** 1 The Pirbright Institute 111636, Pirbright, United Kingdom; 2 The University of Bristol 1980, Bristol, United Kingdom; 3 The Roslin Institute, Easter Bush, Midlothian, United Kingdom; 4 Department of Infectious Disease, Imperial College London 4615, London, United Kingdom; Loyola University Chicago - Health Sciences Campus, Maywood, Illinois, USA

**Keywords:** coronavirus, spike, infectious bronchitis virus, tropism, pathogenicity

## Abstract

Infectious bronchitis virus (IBV), a *Gammacoronavirus*, causes the economically damaging disease of poultry, infectious bronchitis. Development of IBV live attenuated vaccines (LAVs) is dependent on the supply of embryonated hens’ eggs and attenuation through serial passaging, upwards of 80 times. Vaccine development needs to keep pace with IBV evolution; an issue compounded as cross protection between individual IBV serotypes is unpredictable and often limited. Rational attenuation alongside the ability to propagate LAVs in cell culture offers significant advantages in terms of adaptability, cost, and speed. Most field IBV strains exhibit restricted *in vitro* tropism, being unable to replicate in primary or continuous cells without serial passage and adaptation. Beaudette, an attenuated IBV strain, is capable of replication in Vero cells, an ability conferred by the spike (S) glycoprotein. In this study, utilizing a recombinant IBV based on the nephropathogenic IBV strain D388, in which the S sequence is replaced with the equivalent sequence derived from Beaudette, we determine that the Beaudette spike can confer the ability to replicate in Vero cells and Beaudette’s associated attenuated *in vivo* phenotype to a distantly related IBV strain. Assessment of infectious progeny and viral RNA *in vivo* suggests that attenuation was conferred by a tissue tropism-independent molecular mechanism. Analysis of innate immune responses revealed a lower intensity and less inflammatory profile. Our findings support the use of the Beaudette S protein as an avenue for the development of rationally attenuated, cell culture-propagated rIBV vaccines and identify the S protein as a contributor to nephropathogenicity.

**IMPORTANCE:**

Infectious bronchitis virus (IBV), a *Gammacoronavirus* of chickens, causes significant economic losses worldwide. Current live attenuated vaccines (LAVs) are generated through extensive serial passage of virulent IBV strains in specific-pathogen-free (SPF) embryonated hen’s eggs, upwards of 80 passages. This process is slow, dependent on the availability of SPF embryonated eggs, and the molecular basis of attenuation remains poorly understood. Development of rationally attenuated, cell-culture-based IBV vaccines would enable more rapid responses to emerging viral variants/strains. Using a recombinant IBV, we demonstrated the spike glycoprotein from the attenuated IBV strain Beaudette can confer attenuation and the ability to replicate in Vero cells to a distantly related virulent IBV strain. Attenuation was not linked to changes in tissue tropism but was associated with altered host responses. Our research provides new insights into IBV pathogenesis and presents an avenue for rational attenuation that can be exploited for future development of cell-based IBV vaccines.

## INTRODUCTION

Infectious bronchitis virus (IBV), a *Gammacoronavirus*, causes the highly contagious respiratory disease, infectious bronchitis (IB), of poultry. Chickens with IB exhibit respiratory-related clinical signs, including snicking, rales, nasal discharge, as well as watery eyes, lethargy, reduced weight gain, and in laying birds, reduced egg production and quality (1, 2). As a result, IB is considered a risk for food security and is a significant economic burden to poultry industries. Morbidity and mortality are dependent on several factors, including concurrent and/or secondary infections, age, immune status of the chicken, and IBV strain, of which there are many (3). Regardless of host or environmental factors, infection with some strains of IBV results in severe systemic disease including nephritis; these IBV strains are referred to as nephropathogenic (4, 5). Several nephropathogenic strains have emerged in the last 40 years, which have become endemic in several regions across the globe, including B1648 in 1984 (6, 7), QX in 1998 (8–10), and Variant 2 in the mid-1990s to 2000s (11). The molecular mechanism(s) underlying nephropathogenicity are poorly understood; however, excessive stimulation of inflammatory pathways and expression of cytokines, such as the NLRP3 inflammasome, IL-1B, IL-18, and NF-κβ have been implicated (12–14). No specific viral gene has been implicated in nephropathogenicity.

IBV is controlled by biosecurity measures and vaccination, predominantly using live attenuated vaccines (LAVs) generated through serial passage of circulating virulent strains in embryonated hens’ eggs (15), a process that results in unpredictable genetic changes and variable levels of attenuation (16, 17). A fine balance needs to be achieved between attenuation and immunogenicity, as over-passaging can negatively impact vaccine efficacy (17). Few consensus-level changes are acquired, presenting a short molecular route for reversion to virulence through mutations or recombination with field strains (16). Additionally, the process is cumbersome, completely dependent on the supply of specific-pathogen-free (SPF) embryonated hens’ eggs, and time-consuming, as often 80–100 passages are required (15, 17). The process is therefore poorly responsive to emerging strains: an issue compounded by the continual evolution of IBV through genetic drift and/or recombination, paired with the fact that the majority of LAVs are poorly cross-protective between IBV strains of differing serotypes (18–23). Combinations of antigenically different LAVs are used in an effort to induce protection against many circulating serotypes (19, 24–26).

Several avenues have been researched for the development of next-generation IBV vaccines, including subunit vaccine approaches, viral vectors, and more recently, mRNA platforms and transgenic antigen-expressing plants (27–33). Such approaches have centered on the viral-encoded spike (S) protein, which mediates viral entry, as it is the target for the majority of neutralizing antibodies (27, 29, 34–40). While a degree of protection from IBV challenge has been achieved, none have been successfully licensed. Incompatibility with current methods of *en masse* vaccine application via drinking water and/or sprays is a concern, and costs, particularly with mRNA-based vaccines similar to those used against the *Betacoronavirus* severe acute respiratory syndrome coronavirus 2 (SARS-CoV-2) (41, 42), are not compatible with the low-cost production of the poultry industry (43). A more appropriate avenue is the use of reverse genetics to generate rationally designed recombinant IBV (rIBV) LAVs either through modification of attenuated IBV strains or via the rational attenuation of virulent genomes (5, 44–47).

The IBV genome consists of a 27.6 kb positive-sense single-stranded RNA genome that is capped at the 5′ end and polyadenylated at the 3′ end (48, 49). The 5′ two-thirds of the genome contains two large open reading frames, ORF1a and ORF1ab, encoding two large polyproteins, pp1a and pp1ab, with the latter the result of a −1 frameshift (50), that are proteolytically cleaved into 15 non-structural proteins (nsps) (51, 52). These proteins modulate host responses and form the replication-transcription complex (RTC) (51). The 3′ end of the genome encodes four structural proteins: spike (S), envelope (E), membrane (M), and nucleocapsid (N), and the accessory genes 3a, 3b, 4b, 4c, 5a, 5b, and 7 (53–58). Flanking either end of the genome are two untranslated regions (UTR), denoted the 5′ UTR and 3′ UTR, respectively. Several areas of the genome have been targeted for the generation of rationally designed vaccines, including the generation of “spike swap” rIBVs, in which the S gene of one IBV strain has been replaced with the corresponding sequence from another (44, 59–65). The deletion of the accessory genes and mutations in the nsps are also avenues that have been explored (5, 46, 53, 66, 67). The drawback is virus propagation is still dependent on the supply of SPF embryonated hens’ eggs.

Vero cells, a continuous cell line derived from an African green monkey (68), are a widely used cell culture platform for the generation of several vaccines, including those for Ebola, Rabies, Polio, and Yellow Fever (69–72). Although not generally permissive to IBV infection, one strain of IBV, the attenuated Beaudette strain, is capable of replication in Vero cells. However, Beaudette is poorly immunogenic, exhibits highly restricted tropism *in vivo,* and is not suitable as an LAV (44, 73); however, our previous research demonstrated that the ability of Beaudette to replicate in Vero cells is conferred by the S protein (59, 62, 74). Our previous research also identified that the Beaudette S conferred Beaudette’s attenuated phenotype to the virulent IBV M41 (62). Vaccination with the resulting rIBV, M41-Beau(S), elicited 100% protection from a virulent M41 challenge, suggesting (i) that the S protein of Beaudette is immunogenic and (ii) highlighting the dual use of the Beaudette S for both attenuation and viral propagation in Vero cells (62). Both M41 and Beaudette are, however, part of the GI-1 genotype and share 95% amino acid identity in the S protein. The applicability of the Beaudette S as a universal method of attenuation is therefore unknown.

In this study, we aimed to address the question of universal application and used the nephropathogenic IBV strain D388, part of the GI-19 genotype, QX serotype, with only 82% amino acid identity, in the S protein, to Beaudette to (i) establish whether the Beaudette S could be incorporated into a more distantly related IBV backbone, (ii) whether the inserted S gene was genetically stable, (iii) whether both Beaudette’s ability to replicate in Vero cells and its attenuated *in vivo* phenotype would be conferred. During this study, we identified that the rIBV D388-Beau(S) in which the S gene of D388 was replaced with the corresponding sequence from Beaudette was able to replicate in Vero cells to high titers and was attenuated *in vivo*. Tissue tropism *in vivo* was comparable to the pathogenic parental rIBV D388-R and not comparable to Beaudette, suggesting the Beaudette S confers attenuation through a tissue tropism-independent molecular mechanism. Transcriptional profiling of the innate immune response to D388-R and D388-Beau(S) infection highlights differences in the magnitude and breadth of response, suggesting the attenuated phenotype exhibited by D388-Beau(S) may be the consequence of altered host responses. This study highlights the potential universal applicability of the Beaudette S protein in the development of rationally attenuated rIBV LAVs capable of propagation in cell culture and implicates the S protein as a contributing factor in nephropathogenicity.

## MATERIALS AND METHODS

### Ethics statement

All chickens, including those reared for the purpose of providing tissues for cell and organ preparation and those used for *in vivo* experiments, were housed in raised floor pens. Chickens were provided with enrichment which included soft bedding, live feed, dust baths and perches to promote normal animal behavior. All chickens were monitored at least twice daily. Humane endpoints for the *in vivo* study were as follows: (i) sitting alone and not evading capture, in which case the chicken was euthanized immediately; (ii) respiratory distress, for example, excessive gasping, in which case the chicken was euthanized immediately; (iii) rales for 7 days in total, in which case the chicken was euthanized on the beginning of the 7th consecutive day; and (iv) excess drinking for more than 2 days as indicated by a fluid-filled crop, in which case the chicken was euthanized at the beginning of the 3rd consecutive day. Two chickens infected with D388-R reached the humane end point at day 13 post infection (pi), with one bird reaching both endpoints i and ii and the other endpoint i only.

### Cells, tracheal organ cultures, and viruses

Primary chicken kidney (CK) cells were prepared by the Central Services Unit (CSU) at The Pirbright Institute, using kidneys harvested from 2- to 3-week-old, specific-pathogen-free (SPF) Rhode Island Red (RIR) or SPF White leghorn (WL) chickens as previously described (75). Cells were seeded at 0.8 × 10^6^ cells/mL and incubated at 37°C, 5% CO_2_ for 3 days prior to use. Six-well and 12-well plates were seeded with 3 and 2 mL of cells per well, respectively.

Tracheal organ cultures (TOCs) were prepared as previously described using tracheas extracted from the same chickens (76). TOCs were individually seeded in glass tubes and incubated at 37°C in a rolling incubator at 7–8 revolutions per hour (rph) for 3 days before use. Prior to use, ciliary activity was confirmed to exceed 75% using a light microscope.

Embryonated hens’ eggs for use in *in ovo* experiments were purchased from Valo BioMedia, Germany, and incubated at 37°C. Embryonated hens’ eggs for the purpose of hatching and rearing chickens for tissues for cell and TOC preparation were purchased from either Valo BioMedia, Germany, or the National Avian Research Facility, The Roslin Institute, UK.

Vero cells (ECACC: 84113001), a continuous cell line derived from African green monkey kidneys, were provided by CSU at The Pirbright Institute and were maintained using Eagle’s minimum essential media (EMEM) (Sigma Aldrich) supplemented with 10% fetal bovine serum (FBS) (Thermo Fisher), 2 mM L-glutamine (Sigma Aldrich), 100 units/mL penicillin (Gibco), and 100 µg/mL streptomycin (Gibco). DF1 cells (ATCC: CRL-3586), a continuous cell line derived from chicken embryo fibroblasts, were provided by CSU at The Pirbright Institute and were maintained using Dulbecco’s minimum essential media (DMEM) (Sigma Aldrich) supplemented with 10% FBS (Thermo Fisher), 200 mM Hepes (Sigma Aldrich), 100 units/mL penicillin (Gibco), and 100 µg/mL streptomycin (Gibco). All cells were maintained at 37°C, 5% CO_2_.

All viruses have been described previously. The rIBV, D388-R, previously referred to as D388-GGA, is a molecular clone of the nephropathogenic strain D388 (OR813926) (77). The rIBV Beau-R is a molecular clone of the attenuated strain Beaudette-CK (AJ311317) (78). The rIBV D388-Beau(S) contains a chimeric S gene in which the ectodomain of the D388 S gene (nucleotides 20376–23607 within OR813926) has been replaced with the corresponding sequence from Beau-R (nucleotides 20418–23640 within AJ311317) (77). As the coding sequence of nsp16 overlaps with the beginning of S and to maintain interactions with other D388 structural proteins, the signal sequence (SS) (nucleotides 20326–20375 within OR813926), and the transmembrane domain (TM) and cytoplasmic tail (CT) (nucleotides 23608–23796 within OR813926) were all retained as D388-R sequence. All viruses were propagated in 10- to 11-day-old SPF embryonated hens’ eggs, and the quantity of infectious progeny was determined by titration via plaque assays in CK cells or in *ex vivo* TOCs.

### Analysis of the diversity of IBV spike protein sequences

IBV genomes were selected from GenBank using the following criteria: complete genomes, *Coronaviridae* taxid, *Gammacoronavirus galli* species, minimum length 19 kb, maximum length 35 kb. Accessions and sequences were checked to ensure completeness. Sequences were aligned to Beaudette (AJ311317), translated, and the S protein sequence extracted. A maximum likelihood tree was generated from the curated data set using the IQ-TREE web server (AUTO settings) (standard ultrafast bootstrap and SH-aLRT). Amino acid percentage identity to Beaudette (AJ311317) was calculated using the local protein alignments and plotted using ggplot2.

### Next-generation sequencing of D388-Beau(S) stock

D388-Beau(S) virus stock, 4 mL, raised following three passages in CK cells and one passage in embryonated hens’ eggs, was centrifuged at low speed for 10 min, then layered onto 1 mL 10% sucrose cushion and centrifuged at 368,000 × *g*, 4°C, 1 h. The pellet was resuspended in 350 µL RLT buffer containing 2-mercaptoethanol and loaded onto an RNeasy RNA extraction column (Qiagen) with the remaining steps following the manufacturer’s protocol. RNA, 200 ng, was subjected to library preparation using an NEBNext Ultra II directional RNA-Seq kit (NEB, Ipswich, MA). All library preparation was performed in duplicate. The resulting library pools were quantified using a NEBNext Illumina library quantification kit (NEB, Ipswich, MA). Libraries were pooled and loaded onto a 2 × 300 paired-end v3 MiSeq reagent cartridge, on an Illumina MiSeq (Illumina, San Diego, CA). Fastp (79). FastQC (https://www.bioinformatics.babraham.ac.uk/projects/fastqc/) was used to perform initial read quality control (QC) and quality filtering with a minimum quality score of 30 and minimum read length of 100 bp. Quality cutoffs of 30 and trimming of the first 15 bp and final 45 bp were eliminated from reads before mapping to the virus-specific reference sequence using BWA-MEM (80). Alignment pileups were generated with bcftools (v1.10.2), and variants were called from the full pileup using the bcftools multiallelic variant caller. Resulting variant calls were subsequently normalized. For each sample, the corresponding reference sequence was masked with N characters at positions lacking read coverage. Variants with an allele frequency greater than 50%, as determined by the DP4 read depth values, were designated as majority alleles. These majority alleles were then incorporated into the masked reference to generate the final consensus sequence.

### Quantification via titration by plaque assay

CK cells seeded in either 12- or 6-well plates were washed once with phosphate-buffered saline “a” (PBSa), then inoculated with either 250 or 500 µL of 10-fold serially diluted sample, respectively. Following 1-h incubation at 37°C, 5% CO_2_, the inoculum was removed, and an overlay of BES Medium (76) and agar in a 1:1 ratio was added. The plates were incubated at 37°C, 5% CO_2_ for 3 days after which cells were fixed with approximately 2 mL per well of 3.3% formaldehyde in PBSa for 1 h at room temperature. Following fixation, cells were stained with approximately 1 mL per well of 0.1% crystal violet for 20 min at room temperature. The number of virus plaques was counted to determine virus titer as plaque-forming units per mL (PFU/mL). For assessment of plaque morphology, the plaques were photographed, and the diameter of 20 distinctly separated plaques per plate was measured using ImageJ (81).

### Quantification via titration in *ex vivo* TOCs

Samples were 10-fold serially diluted in TOC infection media (62). TOCs, seeded one per glass tube, were washed once with PBSa, and inoculated in replicates of at least three with 1 mL of the diluted sample. Following incubation at 37°C for 6 days at 7–8 rph, the ciliary activity of each TOC was observed using a light microscope. TOCs with less than 5% activity were deemed “dead” and those with greater than 5% deemed “alive”. The titer required to result in 50% reduction in ciliary activity per mL, termed 50% ciliostatic dose/mL (CD_50_/mL), was calculated using the Reed and Muench endpoint calculation (82).

### Assessment of *ex vivo* ciliary activity

TOCs, seeded one per glass tube, were washed once with PBSa. In replicates of ten, TOCs were inoculated with 500 µL TOC infection media containing 10^4^ CD_50_ of rIBV or 500 µl TOC infection media for mock. TOCs were incubated at 37°C, 7–8 rph for 96 h. Using a light microscope, the ciliary activity of every TOC was scored as 0%, 25%, 50%, 75%, or 100% at 24-, 48-, 72-, and 96-h post infection (hpi). The mean average of the 10 replicates was calculated.

### Assessment of *in ovo* replication

Ten-day-old WL SPF embryonated eggs were infected with 100 µL of PBSa containing 10^3^ CD_50_ of rIBV. Following 24-h incubation at 37°C, embryos were culled by refrigeration at 4°C for at least 4 h. The allantoic fluid was harvested, and infectious viral progeny was quantified by titration in *ex vivo* TOCs.

### Assessment of *in vitro* replication

CK, Vero, or DF1 cells seeded in six-well plates were washed twice with PBSa and at confluency were infected with 500 µL BES media containing 10^4^ PFU of rIBV. Following 1-h incubation at 37°C, 5% CO_2_, the inoculum was removed, cells were washed twice with PBSa and 3 mL BES media added. Supernatant was harvested at 1, 8, 24, 48, 72, and 96 hpi and quantified by titration via plaque assay.

### Assessment of *ex vivo* replication

Three TOCs were seeded per tube with each tube defined as one replicate. In replicates of five, TOCs were washed once with PBSa and infected with 500 µL TOC infection media containing 10^4^ CD_50_ rIBV. Following 1-h incubation at 37°C, 5% CO_2_, the TOCs were washed twice with PBSa, and 1 mL TOC infection media was added. TOCs were incubated at 37°C, 7–8 rph for 96 h. Supernatant from one replicate every 24 h up to and including 96 hpi was harvested, and the quantity of viral progeny was quantified by titration in *ex vivo* TOCs.

### Assessment of genetic stability

Ten-day-old WL SPF embryonated eggs in replicates of three or four were infected with 100 µL rIBV diluted 1:10,000 in PBSa. Following 24-h incubation at 37°C, embryos were culled by refrigeration at 4°C for at least 4 h. The allantoic fluid was harvested, diluted 1:10,000 in PBSa, and then used as above to infect embryonated eggs for passage 2. A total of five passages were completed.

RNA was extracted from passage five isolates using an RNeasy kit following the manufacturer’s (Qiagen) protocol for RNA cleanup. RNA was reverse transcribed using Protoscript II Reverse Transcriptase as per the manufacturer’s protocol (New England Biolabs, M0368); reactions were randomly primed (5′-GTT TCC CAG TCA CGA TCN NNN NNN NNN-3′). Nucleotides encoding the S1 and S2 subunit were amplified using *Taq* DNA polymerase as per the manufacturer’s instructions (Invitrogen, 10342020). Primer sets used to amplify S1 were as follows: Beau-R forward (For) (5′-TTATGACTTGGTTTGAAG-3′) and reverse (Rev) (5′-AACTGCCACAAACATACTGC-3′), D388-R For (5′-GAACAACTTAGCTCTGGG-3′) and Rev (5′-GTGAGCTATTGGTTAACTTA-3′), D388-Beau(S) For (5′-TCGGCTTGGACATGTGGTTAT-3′) and Rev (5′-AACTGCCACAAACATACTGC-3′). The following were used to amplify S2: Beau-R For (5′-CATCAAAATCACTAATGG-3′) and Rev (5′-AGGGATCAAATACTTCTGTG-3′), D388-R For (5′-TAAGTTAACCAATAGCTCAC-3′) and Rev (5′-GTTGGCTTCTGTATCATT-3′), and D388-Beau(S) For (5′-CATCAAAATCACTAATGG-3′) and Rev (5′- CTTAGCTCCTGGAACTACTAC-3′). PCR products were sequenced by Sanger sequencing.

### Assessment of *in vivo* pathogenicity

One-day-old SPF RIR chickens were purchased from the National Avian Research Facility, The Roslin Institute, UK. Chickens were divided into randomly assigned groups of 15 that were housed in raised floor pens, with each group in a separate negative pressure HEPA-filtered isolation room. At 8 days old, each chicken was inoculated via the ocular-nasal route with 100 µL PBS or PBS containing 10^4^ PFU or the CD_50_ equivalent of rIBV. Chickens were assessed daily for clinical signs including snicking and rales from day 1–14 post-infection (pi). Chickens were observed by two researchers for 2 min, and the number of snicks observed was recorded and used to calculate the number of snicks per chicken per min. Each chicken was assessed individually for the presence of rales, and the percentage of chickens per group with rales was recorded. On days 4 and 6 pi, five randomly chosen chickens per group were culled via cervical dislocation followed by exsanguination. The birds selected were dictated using the randomizer function in Microsoft Excel. The eyelid, trachea, lung, kidney, and bursa were harvested post-mortem, and sections were stored in either 1 mL PBS or RNAlater (Invitrogen, AM7021). From each chicken, the trachea was sectioned into 10 × 1 mm rings, which were visualized under a light microscope for the assessment of ciliary activity. Ciliary activity was assessed by a person blinded to the sample details and was scored 0, 25, 50, 75, or 100%; the mean of the 10 rings per chicken was calculated. On day 14 pi, the remaining chickens were culled as above.

### Quantification of infectious progeny in tissue samples

Sections of eyelid (0.1 g), trachea (0.22 g), lung (0.16 g), kidney (0.2 g), and bursa (0.1 g) stored in PBS were homogenized in 500 µL of PBSa (containing 100 units/mL penicillin, 100 µg/mL streptomycin, 100 units/mL nystatin) using one 5-mm steel bead (Qiagen) per sample and a TissueLyser II (Qiagen). Homogenized tissue was centrifuged at 17,000 × g for 3 min, and the clarified supernatant stored at −80°C. The quantity of infectious progeny was determined via titration in *ex vivo* TOCs.

### Quantification of IBV-derived RNA in tissue samples

Tissue sections stored in RNAlater were homogenized in 1 mL of Trizol reagent (Invitrogen) using one 5-mm steel bead and a TissueLyser II (Qiagen). Homogenized tissue was centrifuged at 10,000 × *g* for 1 min, the resulting supernatant was transferred to a clean tube, and 200 µL chloroform was added. Following incubation at room temperature for 2 min, samples were centrifuged at 12,000 × *g* for 15 min, 4°C. The upper phase was harvested and mixed with an equal volume of 70% ethanol (Analytical grade, DNase/RNase free). This was loaded onto a RNeasy RNA extraction column (Qiagen) with the remaining steps following the manufacturer’s protocol, including the on-column DNase digestion step.

The quantity of IBV-derived genomic RNA corresponding to the 5′ UTR was quantified using the Luna Universal One-Step RT-qPCR kit (New England Biolabs) using 500 ng of input RNA and primers and probes, at a final concentration of 0.4 and 0.2 µM respectively, as previously described (67, 83). The quantity of IBV-derived RNA corresponding to the N subgenomic mRNA was quantified using the same kit with the following primers: forward 5′-ATATCTATCACACTAGCCTTGCGC-3′ and reverse 5′-TTGCTTTACCGCTTGCCATG-3′, alongside the probe 5′-FAM-TAACAAAGCAGGACAAGCAGAGCCT-TAM-3′, containing a 5′ 6-fluorescein amidite (FAM) fluorescent reporter dye and a 3′ 5-carboxytetramethylrhodamine (Tamra) quencher. Primers and probe were designed using Primer3Plus software version 3.3.0 on default qPCR settings, both ordered from Integrated DNA Technologies, and the probe was purified by high-performance liquid chromatography (HPLC). A plasmid vector pMiniT2.0 containing the sequence amplified by the primers was used as a standard to generate a standard curve to allow absolute quantification of copy numbers. All reactions were carried out in duplicate, with resulting Ct values used to calculate the log_10_ relative RNA copies using the linear equation generated from the standard curve.

### Next-generation sequencing of tissue-derived IBV RNA

IBV-derived RNA was subjected to reverse transcription and second-strand synthesis prior to library preparation using the DNA Prep with enrichment library preparation kit (Illumina). An input of 250 ng of DNA was processed for tagmentation and amplification. Post-tagmentation QC was performed using Tapestation D1000 screentape (Agilent) and Qubit (Life Technologies). Two pools of three libraries were submitted for capture hybridization, which was performed using the 80mer probes (Daicel Arbor) designed for eight serotypes of IBV as described previously (67). Final pooled libraries were eluted after hybridization according to the manufacturer’s instructions. The quality of pooled libraries was checked using Tapestation D1000 screentape (Agilent) and Qubit (Thermo). Final libraries were diluted to 2 nM using Qubit and qPCR (NEBNEXT Illumina library quantification kit, NEB) and loaded according to the manufacturer’s instructions onto a NextSeq 2000 P1 300 cycle reagent kit and flow cell, and spiked with 1% PhiX. Fastq files were taken forward for further analysis. Reads were aligned to their respective reference genomes using the BWA MEM algorithm (BWA v0.7.17) with default parameters. Alignment pileups were generated with bcftools (v1.10.2), and variants were called from the full pileup using the bcftools multiallelic variant caller. Resulting variant calls were subsequently normalized. For each sample, the corresponding reference sequence was masked with N characters at positions lacking read coverage. Variants with an allele frequency greater than 50%, as determined by the DP4 read depth values, were designated as majority alleles. These majority alleles were then incorporated into the masked reference to generate the final consensus sequence.

### Sanger sequencing

PCR products were sequenced by Eurofins, Germany. Sequencing reads were analyzed using Geneious version 2024.0.7.

### Analysis of gene expression via 96.96 IFC Fluidigm dynamic array qPCR

cDNA was synthesized from 500 ng of IBV-derived RNA from trachea and kidneys extracted on days 4 and 6 pi from all five chickens per group using the High-Capacity Reverse Transcription kit (Applied Biosystems) according to the manufacturer’s protocol. cDNA was pre-amplified with TaqMan Pre-Amp Master Mix (Applied Biosystems) and 2.5 µL of a 200-nM mixed pool of primer pairs (Table S1) at 95°C for 10 min, followed by 14 cycles of 95°C for 15 s and 60°C for 4 min. Pre-amplified cDNA was treated with 16 U/µL Exonuclease I (New England Biolabs), at 37°C for 30 min before heat-inactivation at 80°C for 15 min. Pre-amplified, exonuclease-treated cDNA was analyzed through quantitative PCR (qPCR) with the 96.96 IFC Dynamic array (Fluidigm) performed on the BioMark HD instrument (BioMark). Assay mixes were prepared with 2.25 µL 2× Assay Loading Reagent (Fluidigm), 2.5 µL of primer pair mix (1.15 µM), and 0.25 µL low EDTA TE buffer. Sample mixes were prepared with 2.5 µL TaqMan Gene Expression Master Mix (Thermo Scientific), 0.25 µL 20× EvaGreen DNA binding dye (Biotum), 0.25 µL 20× GE Sample Loading reagent (Fluidigm), and 2 µL of pre-amplified, Exonuclease-treated cDNA. Thermal cycling conditions were 50°C for 2 min, 70°C for 30 min, 25°C for 10 min, followed by a hot start step of 50°C for 2 min, 95°C for 10 min, then 30 cycles of 95°C for 15 s, 60°C for 60 s, and fluorescence emission recorded after each cycling step. Raw quantitation cycle (Cq) data were collated with Real-Time PCR Analysis software v 3.1.3 (Fluidigm), setting parameters of quality threshold (0.65), baseline correction (derivative), and Cq threshold method to auto (global). Raw Cq values were processed with GenEx7 (MultiD). Stability of expression of reference genes: TATA box binding protein (TBP), Tubulin alpha chain (TUBA8B), beta-actin (ACTB), beta-glucuronidase (GUSB), glyceraldehyde-3-phosphate dehydrogenase (GAPDH), and ribosomal 28S (r28S) were evaluated with NormFinder (GenEx7), the geometric mean of the most stable (ACTB, GUSB, and TBP) was used for normalization. Technical replicates were averaged, relative quantification was against the maximum Cq value per gene and, transformed to a logarithmic scale.

Significant differentially expressed genes (DEGs) were identified by comparing relative expression values for D388-R or D388-Beau(S) infected trachea and kidney to their corresponding time-matched mock control (*n* = 5/tissue/group/time-point). Group means were compared with two-way *t*-tests adjusted for multiple comparisons with posthoc Bonferroni correction (GenEx7, MultiD).

### Statistical analysis

Unless stated otherwise, statistical analysis was carried out using GraphPad version 10.3.1. Standard deviation and normality of the data were assessed prior to statistical analysis. Details of individual statistical tests are presented in the specific figure legend.

## RESULTS

### The S protein sequence of D388 exhibits a median level of variation compared with the Beaudette strain

**Fig 1:**
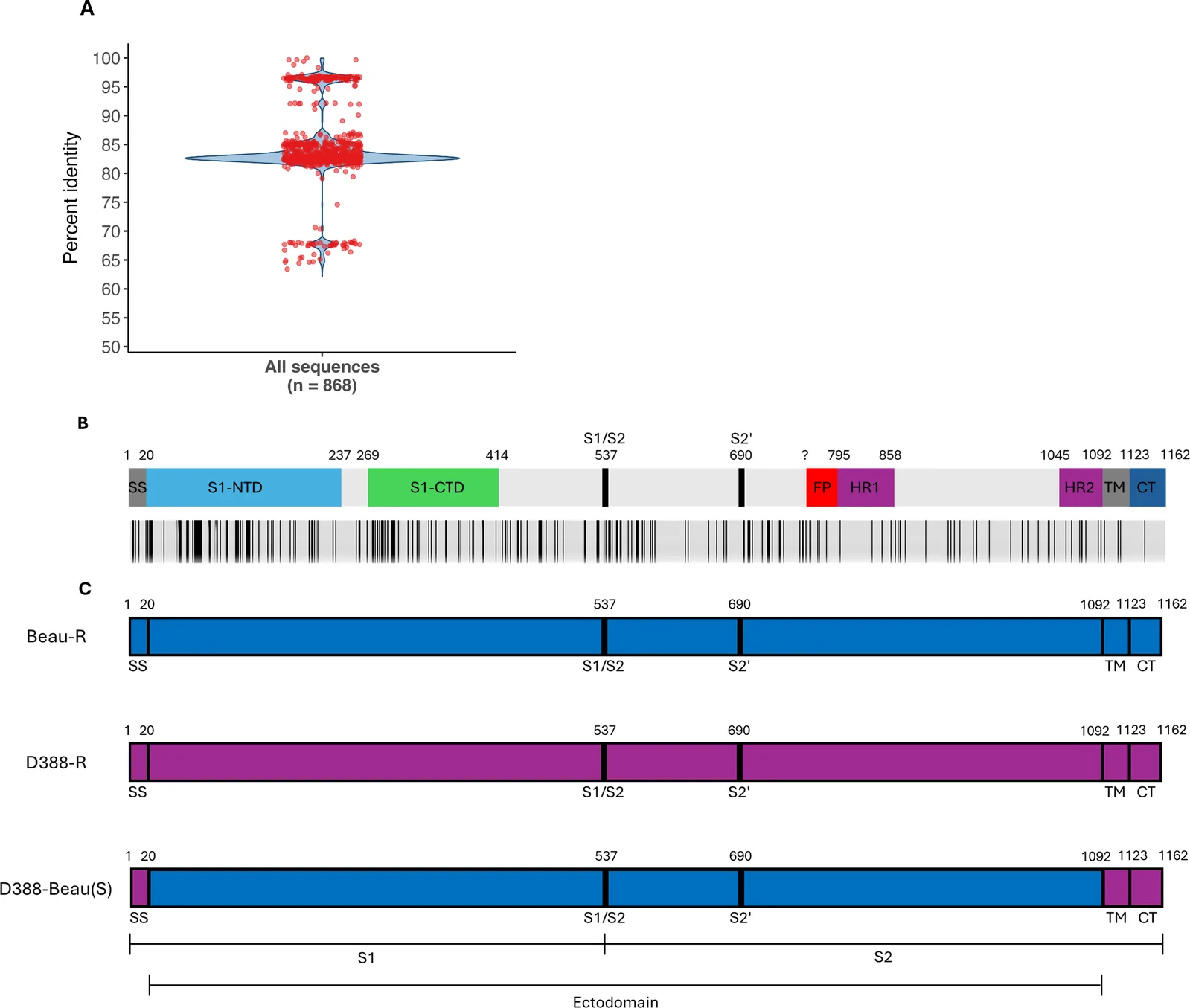
The spike glycoprotein from Beaudette and D388 are genetically diverse. (**A**) All publicly available full-length genome sequences were downloaded from NCBI (www.ncbi.nlm.nih.gov) with duplicate sequences for individual IBV strains removed (*n* = 868). Amino acid sequences relating to the S glycoprotein were aligned with Beaudette (accession number AJ311317) and the percentage identity calculated. (**B**) Schematic showing the location of amino acid differences, highlighted by black lines, between the S proteins of Beaudette (accession number AJ311317) and D388 (accession number OR813926). (**C**) Schematic of the S gene of parental and chimeric rIBVs used in this study. Amino acid numbering is based on the Beau-R sequence. SS, signal sequence; S1-NTD, S1-N terminal domain; S1-CTD, S1-C terminal domain; S1/S2, S1/S2 cleavage site; S2′, S2′ cleavage site; FP, fusion peptide (“?” indicates the exact location is unknown); HR1, heptad repeat 1; HR2, heptad repeat 2; TM, transmembrane domain; CT, cytoplasmic tail.

IBV strains exhibit a high level of diversity, not only in genome sequence but in the severity of IBV associated clinical disease. IBV strains are categorized into genotypes based on the nucleotide sequence of the S1 subunit of the S gene, with both Beaudette and M41 strains members of the GI-1 genotype (3). To investigate S protein amino acid sequence diversity within IBV and identify strains with significantly different S sequence homology to Beaudette, all publicly available IBV sequences marked as full length and over 19 kb were downloaded from NCBI (https://www.ncbi.nlm.nih.gov/). Duplicate sequences for individual IBV strains were removed leaving 868 sequences. Amino acid sequences relating to the S protein were aligned with the Beaudette sequence (AJ311317) and the percentage identity calculated (Fig. 1A). Identities ranged from 63.4% to 100% with the majority of sequences clustering around the median of 82.7%.

The IBV strain D388 is distantly related to both M41 and Beaudette, belonging to the GI-19 genotype. D388 exhibits nephropathogenicity and represents both a strain of economic importance and a genotype that established endemic transmission (84, 85). Sequence analysis highlights 82% amino acid identity between the S protein sequences of Beaudette and D388, equating to 209 amino acid differences: 129 within the S1 subunit and 80 within the S2 (Fig. 1B). This represents the median of observed S diversity of 82.7%. Outside of S, there are variable differences between the Beaudette and D388 genomes, with amino acid identities ranging from 55% to 97% (Table 1). The lowest percentage of amino acid identity was observed in the accessory proteins, with Beaudette exhibiting large truncations in 4b, 4c, and 7.

**TABLE 1:** Beaudette and D388 exhibit amino acid differences across the entire genome^*a*^

Protein	No. of amino acid differences	Percentage identity
nsp2	53	92%
nsp3	198	88%
nsp4	68	87%
nsp5	32	90%
nsp6	36	88%
nsp7	4	95%
nsp8	16	92%
nsp9	2	98%
nsp10	5	97%
nsp11	3	87%
nsp12	44	95%
nsp13	18	97%
nsp14	15	97%
nsp15	23	93%
nsp16	32	89%
Spike	209	82%
3a	9	84%
3b	21	67%
Envelope	13	88%
Membrane	15	93%
4b	10^*b*^	80%
4c	12^*b*^	56%
5a	12	82%
5b	6	93%
Nucleocapsid	40	90%
7	5^*b*^	55%

^*a*^ The sequences of Beaudette (AJ311317) and D388 (OR813926) were aligned, protein sequences translated and compared to assess the amino acid differences and percentage identity.

^*b*^ Highlights large truncations with 4b, 4c, and 7 proteins from Beaudette 44, 21, and 62 amino acids shorter than the D388 equivalent. Percentage identity does not include the truncated sequence.

### Generation of rIBV D388-Beau(S)

Previously, as a proof of principle experiment that a Golden Gate assembly based reverse genetics system could be used to make large gene swaps, the rIBV D388-Beau(S)-GGA was generated (77). This virus, denoted in this paper as D388-Beau(S), is based on the genome of the IBV strain D388 (84) with the nucleotides encoding the ectodomain of the S gene (20376–23607) replaced with the equivalent sequence derived from the molecular clone of Beaudette, Beau-R. While the naming indicates a whole S swap, the S gene of D388-Beau(S) is chimeric (Fig. 1C) as the nucleotides encoding the signal sequence (20326–20375), transmembrane domain (TM) (23608–23703), and cytoplasmic tail (CT) (23704–23796) are derived from D388 in order to (i) preserve the sequence of nsp 16, which overlaps in the signal sequence, and (ii) to preserve the D388 sequence of the TM and CT sequences for maintaining interactions with the other D388 proteins, including E and M (86–88). The sequence of rIBV D388-Beau(S) was confirmed to be as expected via Illumina next-generation sequencing, including the three synonymous mutations, T735A, T891A, and C20907T, made to permit genome assembly. Coverage was 98.6% with an average read depth of 46,000. The only areas of low coverage (below 10 reads) were the 5′ and 3′ ends (nucleotides 0–65 and 27758–28078).

### D388-Beau(S) exhibited Beau-R like replication kinetics

**Fig 2:**
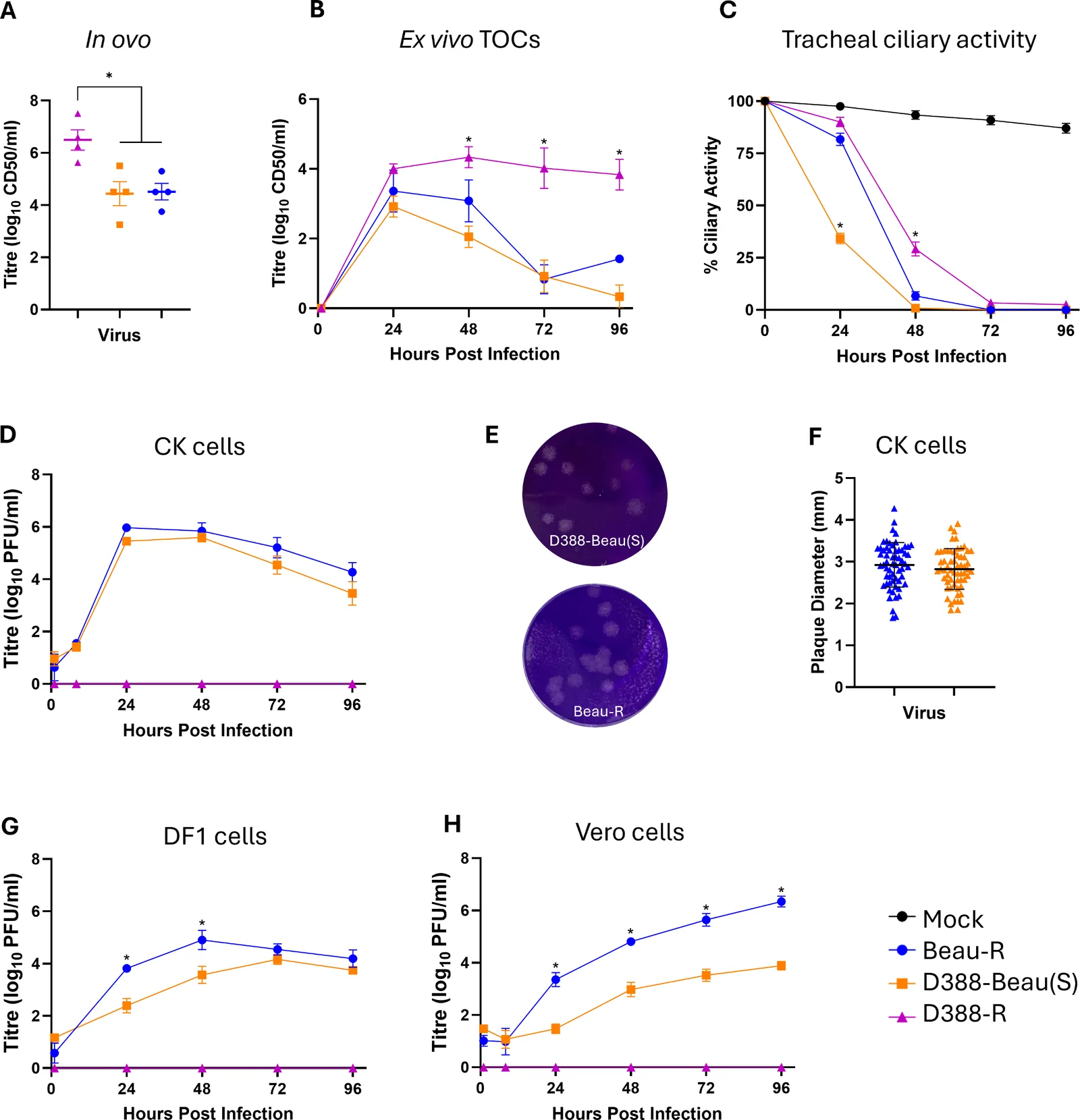
D388-Beau(S) exhibits comparable replication to Beau-R *in ovo, ex vivo,* and *in vitro.* (**A**) Embryonated hen eggs, (**B** and **C**) tracheal organ cultures, (**D **and **F**) CK cells, (**G**) DF1 cells, and (**H**) Vero cells were infected with (**A**) 10^3^ CD_50_, (**B** and **C**) 10^4^ CD_50_, (**D** through **H**) 10^4^ PFU of Beau-R, D388-Beau(S), or D388-R, or mock infected with TOC infection media (**C**). Supernatant (**B**, **D**,** G,** and **H**) was harvested at 24-h intervals, and allantoic fluid (**A**) was harvested at 24 hpi. The quantity of infectious progeny was determined via titration in *ex vivo* TOCs or plaque assay. (**C**) Ciliary activity was assessed using a light microscope with the mean of 10 replicates calculated at each time point. (**E** and **F**) Cells were fixed and stained with crystal violet at 72 hpi, images were taken, and plaque diameter was measured using ImageJ. (**E** and **F**) D388-R was not included in this assay as it is incapable of productive replication on CK cells. (**A** through **C**, **D,** and **F **through **H**) Error bars represent the standard error of the mean of three (**B** through **H**) or four (**A**) independent experiments. Statistical differences, highlighted by an asterisk (*, *P* < 0.05), were assessed by (**A**) one-way ANOVA or (**B**, **C**, **D**,** G,** and **H**) two-way ANOVA. (**B**) Asterisk represents differences between D388-R and both other virus groups. (**C**) Asterisk represents differences between the highlighted time point and all other virus groups. From 24 hpi, ciliary activity in Beau-R, D388-Beau(S), and D388-R infected groups is significantly lower than mock-infected TOCs. (**G** and **H**) Asterisk highlights the difference between Beau-R and D388-Beau(S). From 8 hpi, D388-R is significantly different from all other groups. (**F**) Error bars represent the standard deviation of 20 plaques measured from three independent experiments.

Growth kinetics of D388-Beau(S) and parent viruses D388-R and Beau-R were compared *in ovo*, *ex vivo,* and *in vitro* (Fig. 2). Titers of infectious virus produced by D388-Beau(S) and Beau-R infection *in ovo* (Fig. 2A) and in *ex vivo* tracheal organ cultures (Fig. 2B) were similar, suggesting comparable replication kinetics. *In ovo* and *ex vivo,* titers of both the S donor virus Beau-R and the chimeric virus D388-Beau(S) were significantly lower than the parental strain D388-R (Fig. 2A and B). In *vivo,* IBV infection results in the loss of ciliary activity in the trachea (1, 18). Similarly to both parental viruses, infection of *ex vivo* TOCs with D388-Beau(S) lead to a loss of ciliary activity; however, a more rapid decline in ciliary activity was observed (Fig. 2C).

*In vitro*, the replication kinetics of D388-Beau(S) was assessed in primary CK cells and continuous cell lines, Vero and DF1. In primary CK cells, the titers of D388-Beau(S) and Beau-R are comparable over a 96-h time course, suggesting comparable replication kinetics (Fig. 2D). Plaque morphology is also comparable, as is plaque diameter (Fig. 2E and F). In continuous cell lines, DF1 and Vero, D388-Beau(S) exhibits a comparable pattern of replication to Beau-R; however, titers of D388-Beau(S) are lower from 24 to 96 h (Fig. 2G and H). The parental strain D388-R is unable to productively replicate *in vitro*. The replacement of the S gene with that of Beau-R in D388-Beau(S) has therefore permitted replication *in vitro,* demonstrating the Beaudette S protein can confer tropism to a distantly related IBV genomic backbone.

### The Beaudette S gene sequence in D388-Beau(S) is genetically stable during passage *in ovo*

To understand the compatibility of the Beaudette S with the D388 backbone, the stock of D388-Beau(S) was serially passaged alongside Beau-R and D388-R *in ovo*. A total of five passages were completed in three independent replicates. RNA was extracted from passage five allantoic fluid, reverse transcribed, and the S genes were amplified by PCR, with resulting products sequenced by Sanger sequencing. No mutations were identified in any passaged rIBV, indicating the S gene of D388-Beau(S) is genetically stable.

### Chickens infected with D388-Beau(S) exhibited reduced clinical signs

**Fig 3:**
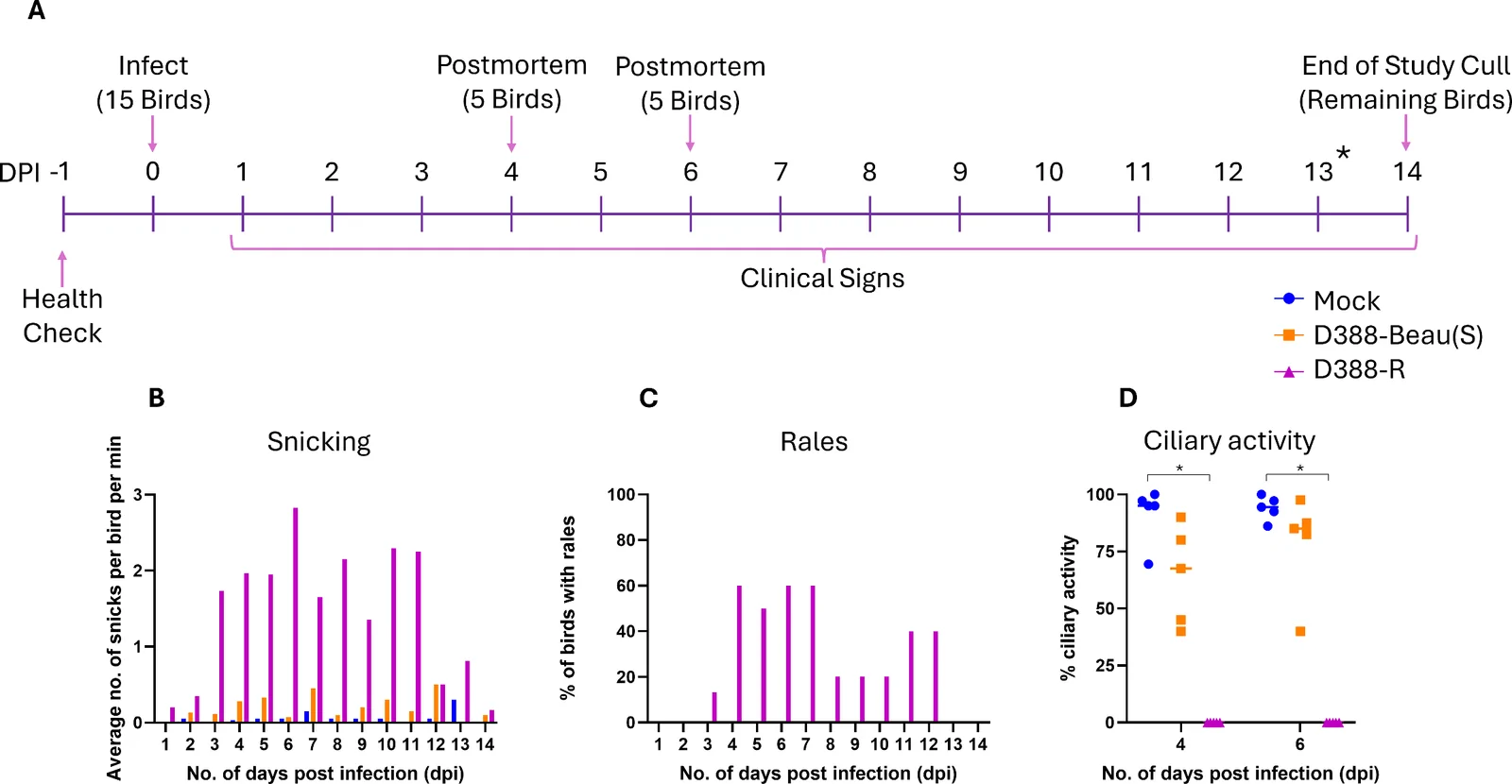
D388-Beau(S) exhibits an attenuated *in vivo* phenotype. (**A**) Chickens were assessed as healthy prior to the study and were randomly assigned into three individually housed groups of 15. At 8 days old, the chickens were mock infected with PBS or infected with 10^4^ PFU of rIBV via the ocular-nasal route. Clinical signs, including snicking and rales, were monitored daily for 14 days pi. On days 4 and 6 pi, five randomly selected chickens were culled, with the remaining chickens culled at the end of the study, day 14 pi, except for two chickens in the D388-R infected group that reached the humane endpoint and were euthanized at day 13 pi (*). Tracheal ciliary activity was observed in harvested trachea on days 4 and 6 pi. (**B**) Chickens were observed by at least two researchers for 2 min, and the number of snicks per chicken per minute was calculated. (**C**) Chickens were individually assessed for rales, with the percentage of chickens positive for rales per group calculated. (**D**) At days 4 and 6 pi, five randomly selected chickens from each group were culled, and the trachea was extracted. The trachea were cut into 10 × 1-mm rings, which were visualised under a light microscope and scored for ciliary activity. The mean ciliary activity per trachea was calculated. Each point represents an individual animal. Statistical significance, highlighted by an asterisk (*, *P* < 0.05), was assessed by a one-way ANOVA.

To determine whether the Beaudette attenuated *in vivo* phenotype was conferred to D388-Beau(S), a pathogenicity experiment was completed (Fig. 3A). Groups of 15, 8-day-old RIR chickens were inoculated via the intraocular and intranasal route, thereby mimicking the natural route of infection, with either the pathogenic control D388-R, D388-Beau(S), or PBS for mock infection. Beau-R was not included in this study, as its attenuated *in vivo* phenotype has been well characterized (44, 73). Clinical signs, including snicking and rales, were monitored every day for 14 days pi. In the mock-infected group, only low levels of snicking and no incidences of rales were observed (Fig. 3B and C). The D388-Beau(S) infected group exhibited levels of snicking much lower than D388-R, more akin to that observed in the mock-infected group (Fig. 3B); no chickens in the D388-Beau(S) group exhibited rales (Fig. 3C). The D388-R infected group exhibited high levels of snicking from days 3 to 11 pi, peaking at day 6 pi (Fig. 3B). Rales were observed from days 3 to 12 pi, with the highest percentages of chickens positive for rales, 60%, at days 4, 6, and 7 pi (Fig. 3C). The reduction in clinical signs suggests that, in comparison to the pathogenic control group, D388-R, D388-Beau(S) exhibited an attenuated phenotype. In support of this, it must be noted that two chickens in the D388-R infected group reached the humane endpoint and were euthanized at day 13 pi. No chickens infected with D388-Beau(S) reached the humane endpoint.

### D388-Beau(S) exhibited an attenuated *in vivo* phenotype

Five randomly selected chickens were culled on days 4 and 6 pi for both the assessment of tracheal ciliary activity for the determination of attenuation and for the analyses of tissue tropism and viral replication. The sampling points were guided by previous research demonstrating that the peak of viral infection in the trachea occurs between days 4 and 6 pi (44, 62, 67, 73, 89). Reductions in tracheal ciliary activity at these time points are indicative of infection with pathogenic IBV. As expected, tracheas extracted from chickens infected with D388-R, the pathogenic control, showed complete cessation of ciliary activity, ciliostasis, on both days 4 and 6 pi (Fig. 3D). Tracheas extracted from mock-infected chickens exhibited ciliary activities of 70% to 100% and 86% to 100% on days 4 and 6 pi, respectively. Tracheas extracted from chickens infected with D388-Beau(S) exhibited a range of ciliary activities from 40% to 90% on day 4 pi and 40% to 98% on day 6 pi. On both days 4 and 6 pi, the majority of chickens, 3/5 and 4/5, respectively, from the D388-Beau(S) group were comparable to those from the mock-infected group (Fig. 3D). This alongside the absence of IBV specific clinical signs (Fig. 3B and C) is suggestive that D388-Beau(S) exhibits an attenuated phenotype *in vivo*.

### D388-Beau(S) exhibited comparable *in vivo* tissue tropism to the pathogenic control D388-R

To investigate whether the attenuated *in vivo* phenotype exhibited by D388-Beau(S) was associated with reduced replication and/or restricted tropism, RNA was extracted from tissues harvested on days 4 and 6 pi, which represented both primary and secondary sites of replication. The levels of IBV-derived genomic RNA were quantified via quantitative real-time PCR using well-characterized primers and probes targeting the 5′ UTR (67, 77, 83). As IBV RNA persistence has been reported, and studies investigating SARS-CoV-2 have indicated that the presence of genomic RNA may not represent active replication and that the detection of sgmRNA is more correlative with the production of infectious virus particles (90–94), IBV-derived RNA encoding N subgenomic mRNA (sgmRNA) was also quantified. Primers and probes for the detection of N sgmRNA were designed using Primer3Plus (Version 3.3.0) and tested to ensure a single amplicon was produced that was subsequently sequence-verified.

**Fig 4:**
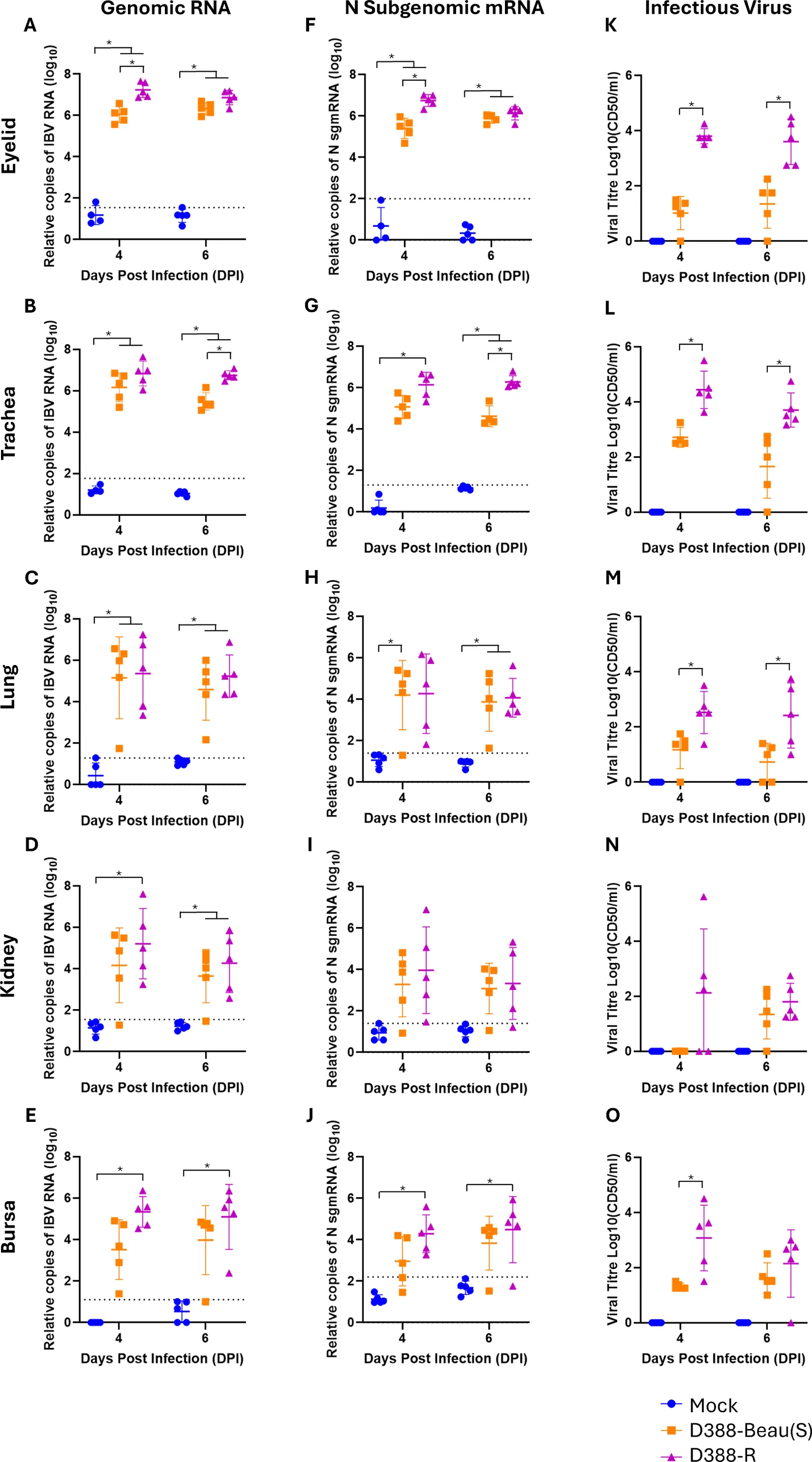
D388-Beau(S) exhibits comparable tissue tropism *in vivo* to the pathogenic control D388-R. RNA extracted at days 4 and 6 pi, from the eyelid (**A** and **F**), trachea (**B** and **G**), lung (**C** and **H**), kidney (**D** and **I**), and bursa (**E** and **J**), was assessed for the quantity of IBV-derived RNA by quantitative RT-PCR using primers and probes targeting the 5′ UTR (**A **through **E**) or the N sgmRNA (**F **through **J**). The quantity of infectious virus in homogenized eyelid (**K**), trachea (**L**), lung (**M**), kidney (**N**), and bursa (**O**), extracted on days 4 and 6 pi, was determined by titration in *ex vivo* TOCs. (**A** through **O**) Each point represents an individual animal, with error bars showing standard deviation. Statistical significance, represented by an asterisk (*, *P* < 0.05), (**A** through** J**) was assessed by a one-way ANOVA with a Tukey test for multiple comparisons or (**K** through **O**) was assessed between D388-R and D388-Beau(S) using an unpaired two-tailed *t*-test.

IBV-derived RNA was detected in all tissues harvested from animals infected with D388-R and D388-Beau(S), suggesting comparable *in vivo* tropism between both rIBVs (Fig. 4A through J). The quantity of IBV-derived genomic RNA was comparable on both days 4 and 6 pi in lung (Fig. 4C), kidney (Fig. 4D), and bursa (Fig. 4E): tissues representing secondary sites of IBV replication (1). Reduced levels of IBV-derived genomic RNA were observed in chickens infected with D388-Beau(S) in the eyelid tissue harvested on day 4 pi and in the trachea on day 6 pi (Fig. 4A and B). These results were mirrored through the quantification of N sgmRNA (Fig. 4F through J).

To investigate how correlative the quantity of IBV-derived RNA is to the presence and quantity of infectious virus, eyelid, trachea, lung, kidney, and bursa tissues were homogenized, and the resulting tissue-derived supernatant was titrated in *ex vivo* TOCs. As expected, no infectious virus was detected in tissues extracted from mock-infected chickens (Fig. 4K through O). Infectious virus was detected in all tissues extracted from D388-Beau(S) and D388-R infected chickens, suggesting no differences in overall tissue tropism. In all tissues analyzed, titers of D388-Beau(S) were lower in comparison to D388-R on both days 4 and 6 pi (Fig. 4K through O), suggesting productive replication of D388-Beau(S) is reduced. The presence of infectious virus and IBV-derived RNA in comparable tissues, corresponding to D388-Beau(S) and D388-R, suggests that the attenuated phenotype exhibited by D388-Beau(S) is facilitated by a tissue tropism-independent molecular mechanism.

### *In vivo* tropism does not appear to be the result of sequence changes in D388-Beau(S)

To determine whether the comparable *in vivo* tissue tropism observed between D388-Beau(S) and D388-R was the result of sequence changes, RNA derived from kidney tissue from three chickens per group at day 6 pi was sequenced using Illumina sequencing with probe enrichment (67). The samples chosen contained the highest quantity of IBV-derived RNA, as determined by qRT-PCR (Fig. 4D), in order to maximize the likelihood of full genome coverage. Sequencing reads were aligned to the corresponding reference sequence, either D388-R (Accession number: OR813926) or D388-Beau(S). Over 98.6% coverage for all six samples was achieved, with mean read depth ranging from 164 to 12,225 (Fig. S1). None of the D388-R sequences exhibited any non-synonymous mutations. One D388-R sequence contained a single point mutation, A25500T; while synonymous, it is located within the transcriptional regulatory sequence for gene 5. Of the three D388-Beau(S) sequences, one contained a synonymous mutation within nsp14, C18521T, and one contained a non-synonymous mutation in S, T20579G, resulting in I52S within the S1-NTD. This mutation was observed in 37% of reads, with 137 reads containing the unmutated sequence.

### Innate immune responses in the trachea elicited by D388-Beau(S) are functionally diverse and resolve by day 6 pi

**Fig 5:**
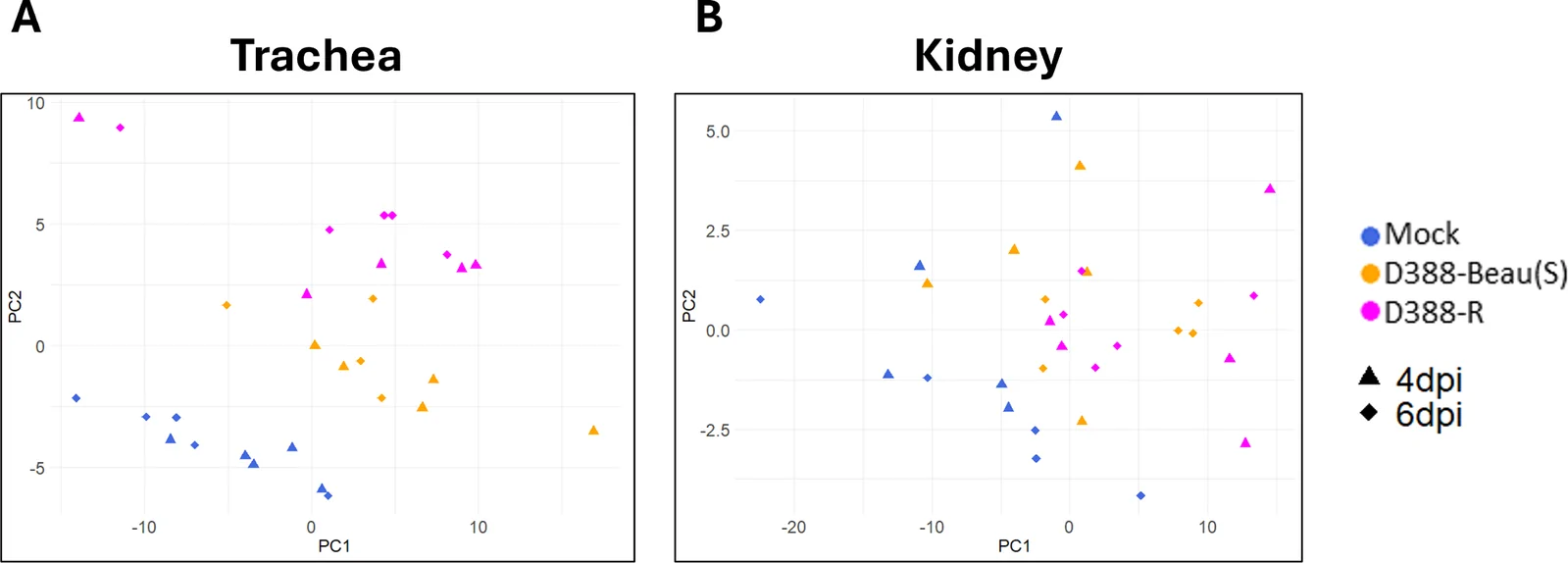
Principal component analysis of the global gene expression of viral innate immune-related genes. RNA extracted from (**A**) the trachea and (**B**) kidney from all chickens culled on days 4 and 6 pi was analyzed by a Fluidigm qPCR array.

**Fig 6:**
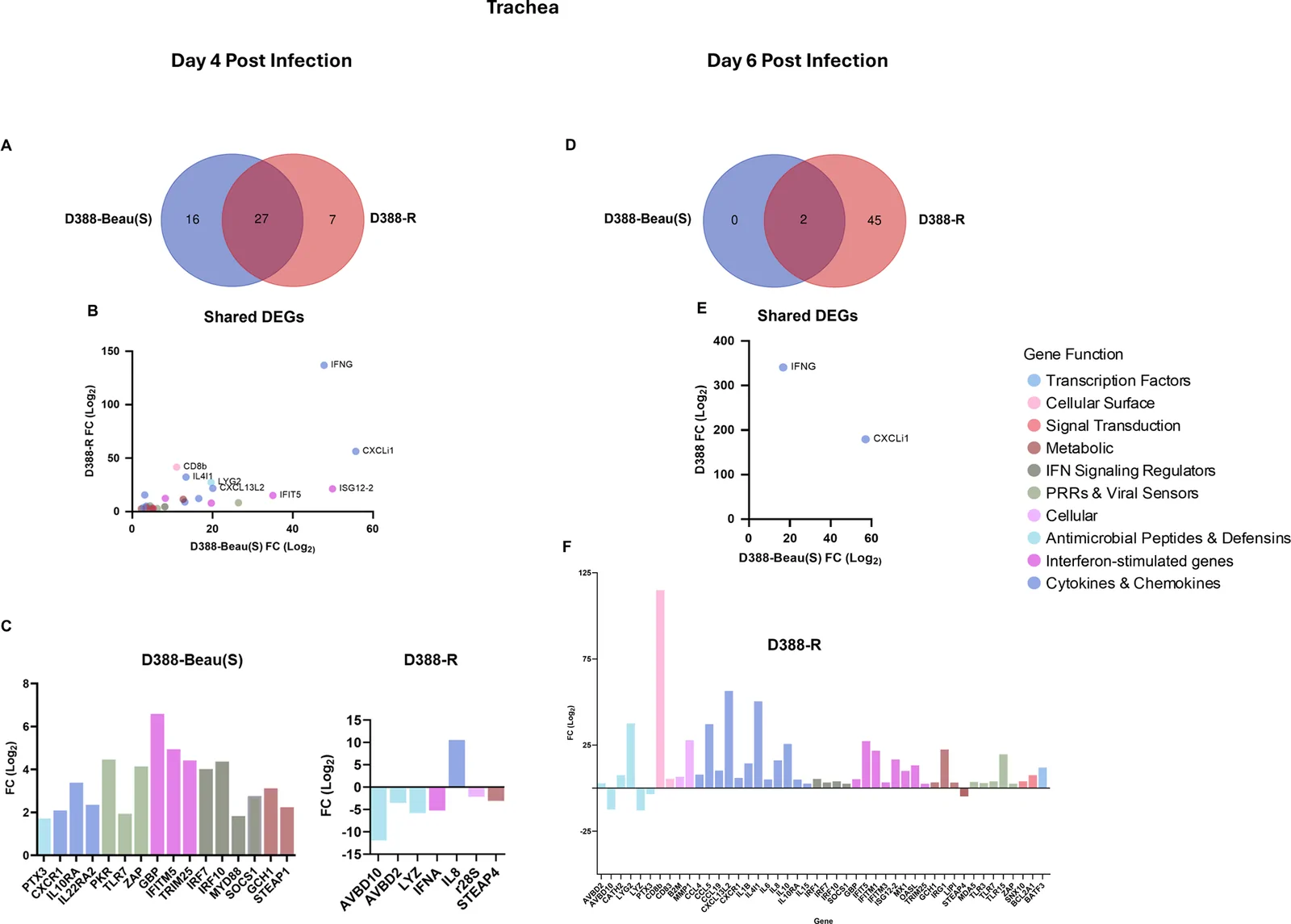
D388-R and D388-Beau(S) trigger both shared and strain-specific transcriptional responses in the trachea. Significant DEGs were identified in the trachea at day 4 (**A** through **C**) and day 6 (**D** through **F**) pi with D388-R or D388-Beau(S) with 96.96 IFC Fluidigm qPCR. (**A**) Venn diagram showing the number of DEGs in both infection groups (27 genes) and uniquely regulated in D388-Beau(S) (16 genes) or D388-R (seven genes). (**B**) Fold change (FC) values of DEGs, via comparison to appropriate mock, classified by function, shared between D388-R and D388-Beau(S). (**C**) Functional classification of genes uniquely regulated in each infection group. (**D**) Venn diagram showing the number of significant DEGs in both groups (two genes) and unique in D388-R (45 genes). (**E**) FC values of DEGs, via comparison to appropriate mock, shared between D388- and D388-Beau(S)-infected groups, with genes classified by functional categories. (**F**) Functional classification of genes uniquely regulated (*n* = 5/tissue/group).

As D388-Beau(S)-infected chickens showed no signs of kidney damage, and previous studies have linked innate immune signaling and inflammatory cytokines with nephropathogenicity (12–14), we investigated innate immune responses in the trachea and kidney following infection with D388-R and D388-Beau(S) using tissue-derived RNA analyzed by a Fluidigm Dynamic Array qPCR (95, 96). Principal component analysis (PCA) of tracheal samples showed that global gene expression clustered according to infection status (Mock, D388-Beau(S), D388-R) (Fig. 5A). At day 4 pi, both D388-R and D388-Beau(S) infection induced a shared transcriptional response, comprising 27 significantly upregulated genes. These genes were associated with interferon-related pathways (*ISG12-2, IFIT5*), cytokine signaling (*CXCLi1, IFNG, IL4I1, CXCL13L2, CD8b*), and pathogen-sensing functions (Fig. 6A and B), indicating activation of a conserved innate response. Despite this shared response, strain-dependent differences in expression magnitude and gene composition were observed (Fig. 6A and C), including variation in *IFNG*, *CD8b*, and *IL4I1* expression. D388-R infection was associated with a limited number of uniquely regulated genes (*n* = 7), whereas D388-Beau(S) induced a broader strain-specific profile, including increased regulation of interferon signaling mediators (*TRIM25, IFITM5, GBP, MYD88, IRF10, IRF7*) and pattern-recognition receptors (*ZAP, TLR7, PKR*) (Fig. 6C). By day 6 pi, transcriptional responses in the trachea were predominantly associated with D388-R infection, with 47 significant DEGs identified, of which 45 were specific to D388-R (Fig. 6D and F). Only *IFNG* and *CXCLi1* remained commonly regulated between strains, with higher expression in D388-R-infected chickens (Fig. 6E). No D388-Beau(S)-specific DEGs were identified at this timepoint. D388-R-specific genes spanned multiple functional categories, including interferon-stimulated genes, cytokines and chemokines, pattern-recognition receptor signaling components, and antimicrobial effectors (Fig. 6F).

### Innate immune responses in the kidney elicited by D388-R are more pro-inflammatory and anti-microbial than those elicited by D388-Beau(S)

**Fig 7:**
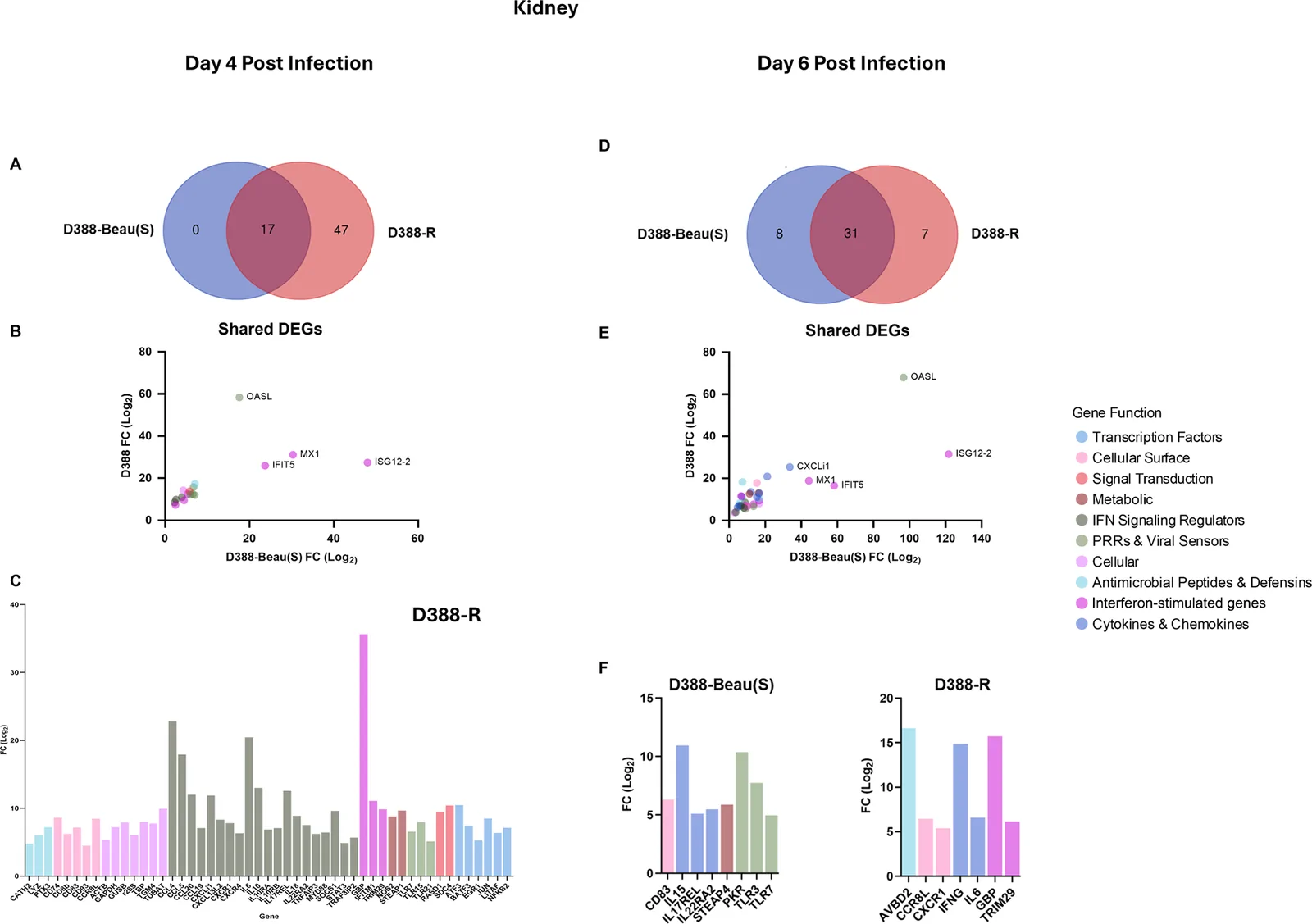
Transcriptional responses in the kidney at day 4 pi are skewed towards D388-R infection but converge at day 6 pi. Significant DEGs were identified in the kidney at day 4 (**A** through **C**) and day 6 (**D** through **F**) pi with D388-R or D388-Beau(S) with 96.96 IFC Fluidigm qPCR. (**A**) Venn diagram showing the number of significantly regulated DEGs in both groups (17 genes) and uniquely regulated in D388-R (47 genes). (**B**) FC values of significantly regulated genes, via comparison to appropriate mock, shared between D388-R- and D388-Beau(S)-infected groups, with genes classified by functional categories. (**C**) Functional classification of genes uniquely regulated in each infection group. (**D**) Venn diagram showing the number of significant DEGs in both infection groups (31 genes) and those uniquely regulated in D388-R (seven genes) and in D388-Beau(S) (eight genes). (**E**) FC values of significantly regulated genes, via comparison to appropriate mock, shared between D388-R- and D388-Beau(S)-infected groups, with genes classified by functional categories. (**F**) Functional classification of genes uniquely regulated in each infection group (*n* = 5/tissue/group).

In the kidney, PCA indicated that global gene expression profiles were variable and did not clearly separate between D388-R- and D388-Beau(S)-infected chickens (Fig. 5B). At day 4 pi, the transcriptional response was skewed towards D388-R infection with 17 DEGs shared between D388-R and D388-Beau(S) and 47 uniquely associated with D388-R (Fig. 7A). The shared response included upregulation of *OASL* and canonical ISGs (*IFIT5, ISG12-2, MX1*) (Fig. 7B). D388-R infection was associated with marked upregulation of three interferon-regulatory and ISG transcripts (Fig. 7C). By day 6 pi, responses became more similar between strains, with 31 genes significantly regulated in both groups and relatively few strain-specific DEGs (eight for D388-Beau(S), seven for D388-R) (Fig. 7D). Shared ISGs (*ISG12-2, OASL, MX1, IFIT5*) remained highly expressed in both groups (Fig. 7E). At this timepoint, D388-Beau(S) infection showed increased expression of pattern-recognition receptors (*TLR7, TLR3, PKR*) and cytokine-associated genes (*IL22RA2, IL17REL, IL15*) (Fig. 7F). In contrast, D388-R infection was associated with increased expression of additional ISGs (*TRIM29, GBP*), pro-inflammatory mediators (*IL6, IFNG*), and avian β-defensin-2 (Fig. 7F).

## DISCUSSION

The development of IBV LAVs is dependent on the supply of SPF embryonated hens’ eggs and attenuation of virulence through serial passaging. As more IBV strains emerge and are identified, vaccine development lags behind, unable to keep pace with IBV evolution (43), an issue compounded by the unpredictable and often limited cross-protection between serotypes (18–23). Rational attenuation alongside the ability to propagate LAVs in cell culture offers significant advantages in terms of adaptability, cost, and speed. *In vitro* replication of IBV is highly restricted, with the majority of field strains only able to be propagated *in ovo* and *in vivo,* impeding the ability to rationally attenuate IBV. However, the highly attenuated Beaudette strain of IBV is able to replicate in Vero cells (62, 74), a cell line licensed and used for vaccine production (69–71). Beaudette’s ability to replicate in Vero cells has been mapped to the S glycoprotein (59, 62, 74). Other IBV strains, following serial passage, have gained the ability to replicate in Vero cells, with this tropism being linked to mutations within either the S1 or S2 subunits, some of which are Beaudette-like (97–99). Recently, an IBV strain belonging to the GVII genotype was isolated, which is capable of replication in Vero cells without serial passage in cell culture; however, the amino acid residues responsible for this tropism are yet to be elucidated (100).

The aim of this study was to build on our previous research and to establish whether the Beaudette S has the potential to offer a universal methodology for both rational attenuation and *in vitro* propagation for any IBV strain, endemic or emerging. Our previous work utilized highly related strains belonging to the same genotype (GI-1) and serotype (Mass), which have high S protein amino acid homology. It was unknown whether the presence of a heterologous S gene, which differed more significantly from the backbone strain, would be both genetically stable and confer both Beaudette’s cell tropism and attenuated *in vivo* phenotype to the resulting recombinant IBV. The S protein of IBV is diverse, with our analysis of spike sequences from publicly available full-length genome IBV sequences highlighting 63% to 100% amino acid homology to Beaudette, with the majority of sequences clustering around 82.7% (Fig. 1A). D388, with an S amino acid homology to Beaudette of 82% is an appropriate representative of known IBV strains. It must be noted that our search criteria excluded partial IBV sequences and therefore may not represent all S sequences from all currently circulating IBV strains.

Assessment of the replication kinetics of rIBV D388-Beau(S) demonstrated that the presence of a diverse heterologous S glycoprotein did not negatively impact viral replication (Fig. 2). This was supported via serial passaging, in which no clearly identifiable compensatory mutations were observed in the S gene sequence. The ability to replicate in Vero and DF1 cells was also conferred by the Beaudette S protein (Fig. 2G and H), supporting previous research that the IBV S glycoprotein is a determinant of tropism (59, 62, 74, 101, 102) and may offer a universally applicable methodology for IBV propagation in Vero cells (59, 62, 74). Titers of D388-Beau(S), however, did not reach the same levels as the parental rIBV Beau-R in Vero cells and were on average two orders of magnitude lower at each assessed time point (Fig. 2H). This is unlikely to be the result of incompatibility between the Beaudette S and D388 backbone sequences, as replication in primary CK cells (Fig. 2D) is comparable between D388-Beau(S) and Beau-R, demonstrating the Beaudette S glycoprotein can confer a complete tropism shift; D388-R is incapable of replication in primary CK cells. Sequence differences outside of the S gene are more likely to account for the overall replication difference observed between D388-Beau(S) and Beau-R. In support of this, whole genome sequencing of Vero-adapted rIBV H120 and IBV strain tl/CH/LDT3/03 highlighted mutations outside of S, including those in 1ab (97, 103). IBV strain SD, when adapted to another mammalian cell line, BHK, highlighted mutations within S, alongside mutations in nsp4 and E, which facilitated replication in BHK cells (104).

The demonstration of the reduction in clinical signs, as well as the retention of ciliary activity (Fig. 3B through D), indicated that D388-Beau(S) exhibited an attenuated *in vivo* phenotype, suggesting that the Beaudette S gene offers a promising avenue for the rational attenuation of IBV. Previous research demonstrated that the replacement of the S gene of the pathogenic M41 strain (genotype GI-1) with that of the pathogenic 4/91 strain (genotype GI-13) resulted in a rIBV with a pathogenic *in vivo* phenotype (62). The 4/91 S sequence shares 81.7% amino acid identity with that of M41, comparable to the 82% shared identity between the S glycoproteins of D388 and Beaudette. It is therefore reasonable to conclude that the attenuated phenotype observed for D388-Beau(S) can be specifically attributed to the presence of the Beaudette S glycoprotein, which supports our previous findings utilizing the pathogenic M41 backbone (62). This observation also further highlights that the S glycoprotein of IBV is a pathogenicity factor (53, 63, 101), in line with other coronaviruses including transmissible gastroenteritis virus (TGEV), murine hepatitis virus (MHV), and SARS-CoV-2 (105–109). It must be noted, however, that replacement of S, S1, or S2 in Beaudette or S in the vaccine strain H120, with an equivalent sequence derived from pathogenic IBV strains does not confer a pathogenic phenotype to the resulting rIBVs (44, 60, 61, 64, 65). The Beaudette backbone exhibits a temperature-sensitive (*ts*) replication phenotype (73), and likely, similarly to H120, contains multiple attenuating lesions. The replacement of S gene sequence with that derived from pathogenic strains is insufficient to overcome this attenuation and/or *ts* phenotype, indicating that the contribution of S to pathogenicity is dependent on its genomic context. Supporting this, a recent study demonstrated that identical mutations within the S2′ cleavage site of S can have markedly different effects on pathogenicity and tissue tropism depending on the viral strain (110).

The Beaudette strain of IBV has been well characterized as having restricted *in vivo* tropism, with replication of rIBV Beau-R, a molecular clone of Beaudette-CK (78), limited to the beak tissue containing the nasal turbinates (5, 44). D388-Beau(S) exhibited comparable tissue tropism to the nephropathogenic D388-R, with both infectious virus and IBV-derived RNA isolated from tissues representing both primary and secondary sites of replication (Fig. 4). This suggests that the restricted tropism observed during Beaudette infection is not simply mediated by the S glycoprotein and that the attenuation conferred by the Beaudette S glycoprotein is via a tissue tropism-independent molecular mechanism. This is an interesting observation, as although relatively few studies have examined the effects of modifying S in pathogenic genomic backgrounds, changes in pathogenicity have generally been associated with corresponding alterations in tissue tropism (102, 110–112). Our findings therefore suggest tissue tropism and pathogenicity are not directly linked and furthermore suggest that nephropathogenicity is not solely defined by the ability of IBV to replicate within the kidney. To date, research into IBV nephropathogenesis has largely focused on characterizing the differential host immune responses elicited by nephropathogenic strains (12, 14, 113–115), rather than viral genetic determinants that contribute. By removing the D388 S glycoprotein from D388-Beau(S) and replacing it with the S sequence from an attenuated, non-nephropathogenic IBV strain, our study implicates S as a factor in nephropathogenicity and supports a role for spike-mediated mechanisms in disease pathogenesis beyond tissue tropism alone.

Transcriptional profiling of tracheal and kidney RNA from infected chickens identified strain-specific differences in host innate immune responses. In the trachea, both D388-R and D388-Beau(S) induced a shared interferon-associated response early during infection; however, D388-Beau(S) elicited a broader and more functionally diverse transcriptional profile, including enhanced regulation of PRRs, IFN signaling and cytokine production (Fig. 6). In contrast, D388-R infection was associated with a more focused, early response that progressed to a dominant and sustained upregulation of interferon-stimulated genes, inflammatory cytokines, and antimicrobial effectors by day 6 pi, consistent with a heightened anti-viral and pro-inflammatory state (Fig. 6). Although responses partially converged at later timepoints, D388-R infection maintained elevated expression of ISGs and pro-inflammatory transcripts in both the trachea and kidney, consistent with prolonged innate immune activation. In the kidney, D388-R induced a more pronounced pro-inflammatory and anti-microbial transcriptional profile, whereas responses to D388-Beau(S) were comparatively delayed and less inflammatory. A simple explanation for the differences observed is variations in replication kinetics; however, whilst D388-Beau(S) exhibited lower titers of infectious virus in both the trachea and kidney, quantities of IBV-derived RNA were comparable to D388-R (Fig. 4). Differences observed in the innate immune responses therefore cannot solely be attributed to the reduced viral titres of D388-Beau(S), as viral RNA is recognized by host pattern-recognition receptors as a pathogen-associated molecular pattern and consequently is a potent activator of innate immune signaling pathways (116, 117). Collectively, these findings support an association between sustained innate immune activation and the increased virulence of D388-R, while D388-Beau(S) infection is characterized by a broader but more transient and regulated response, consistent with delayed resolution of early innate activation. These findings are supported by other studies using nephropathogenic IBV strains, which have shown that toll-like receptors (TLRs) recognize the IBV S glycoprotein, producing *IL1B*, *IL6*, *IL18*, *NFκβ*, and *IFNγ*, which associate with tracheal and kidney lesions (12, 13, 113, 115, 118–122). Single-cell RNA sequencing of chickens infected with the nephropathogenic SD strain, genotype GI-19, comparing infected and uninfected subpopulations of tracheal and kidney cells revealed that *IL1B, IL18, IRF7,* and *IRF9,* among others, were upregulated in the infected kidney and tracheal epithelial cells in comparison to uninfected subpopulations (123), further supporting our findings from a tissue-wide analysis. SARS-CoV, SARS-CoV-2, MHV, and human coronavirus (HCoV)-229E S have also been shown to interact with *TLR4,* which activates an abnormal immune response characterized by increased *IL1B* and *IL6* in a MYD88-dependent manner (108, 124).

In summary, this study further contributes to our understanding of viral factors involved in IBV pathogenicity, specifically highlighting a potential contributing role of S in nephropathogenicity. Our study also highlights that the S gene from the laboratory-adapted Beaudette strain of IBV can confer both an attenuated *in vivo* phenotype and the ability to replicate in Vero cells to a distantly related, highly pathogenic IBV strain. The attenuated *in vivo* phenotype conferred occurs in a tissue tropism independent manner, with the resulting rIBV eliciting a less severe and delayed innate immune response. Genetic stability of the heterologous S gene is observed *in vivo* and *in ovo*. The Beaudette S gene offers a potential universally applicable avenue for the development of rationally attenuated recombinant IBV vaccines that can be produced in cell culture, both reducing the reliance on embryonated hens’ eggs and accelerating responsiveness to emerging and endemic IBV strains and variants.

## Data Availability

All experimental data required for the conclusions discussed are present in this paper. All genome sequences used are publicly available.

## SUPPLEMENTAL MATERIAL

Fig. S1 jvi.01103-26-s0001.tif Genome coverage plots of whole genome sequencing.

Supplemental legend jvi.01103-26-s0002.docx Legend for Fig. S1.

Table S1 jvi.01103-26-s0003.xlsx Details of primers for Fluidigm.

ASM does not own the copyrights to Supplemental Material that may be linked to, or accessed through, an article. The authors have granted ASM a non-exclusive, world-wide license to publish the Supplemental Material files. Please contact the corresponding author directly for reuse.
