# Supplementary material for: Spike protein derived from an apathogenic IBV strain confers attenuated phenotype to a nephropathogenic IBV strain
Source: J Virol. 2026 Sep 10;100(10):e01103-26. doi: 10.1128/jvi.01103-26 (PMC13712957; doi:10.1128/jvi.01103-26)
Supplement: Supplementary file 1 [file jvi.01103-26-s0002.docx]

**Supplemental figure 1: Coverage plots of Next-generation Sequencing.** Coverage plots of whole IBV genome sequencing of RNA isolated from allantoic fluid (A) or the kidneys of three chickens infected with D388-R (B-D) or D388-Beau(S) (E-G). Dotted line indicates the mean number of reads across the genome.
